# Long-term treated intensive care patients outcomes: the one-year mortality rate, quality of life, health care use and long-term complications as reported by general practitioners

**DOI:** 10.1186/s12871-015-0121-x

**Published:** 2015-10-12

**Authors:** Simone Steenbergen, Saskia Rijkenberg, Tamara Adonis, Gerda Kroeze, Ilse van Stijn, Henrik Endeman

**Affiliations:** Department of Intensive Care, Onze Lieve Vrouwe Gasthuis, PO box 95500, 1090 HM Amsterdam, The Netherlands

**Keywords:** Intensive care unit, One-year mortality, Health related quality of life, Home return, Use of healthcare, Long-term complications

## Abstract

**Background:**

The purpose of this study was to determine the one-year mortality rate and its predictors regarding long-term intensive care-treated patients together with their health-related quality of life (HRQL), place of living, healthcare use and long-term complication characteristics after intensive care unit (ICU) discharge.

**Methods:**

A retrospective cohort study was performed in a 20-bed mixed ICU. The patients that were treated for more than 72 h between 2007 and 2012 were included in this study. The one-year mortality rate was calculated, and the characteristics of the ICU survivors that died within one year after ICU discharge were further analysed. For all patients, the Dutch version of the SF-36 questionnaire was used to assess their current HRQL. The results were compared with a normal population. Additionally, patients were questioned about their place of living, and their general practitioners (GPs) were questioned about the patients’ possible long-term complications.

**Results:**

Seven hundred and forty patients were included in this study, and their one-year mortality rate was 28 %, of which half died within the first week after ICU discharge. The one-year mortality rate predictors included age at the time of ICU admission, APACHE IV-predicted mortality score, number of comorbidities and ICU re-admissions. The ICU survivor HRQL was significantly lower compared with the normal population. Half of the patients did not return to their pre-hospital place of living, and numerous possible long-term complications were reported, particularly decreased tolerance, chronic fatigue and processing problems of relatives.

**Conclusions:**

One-year mortality rate of long-term ICU-treated patient was 28 %, and this was predicted by age, disease severity, comorbidities and ICU re-admissions. The ICU survivors reported a lower HRQL, and a minority of these patients returned home directly after hospital discharge; however, GPs reported numerous possible long-term complications.

## Background

Intensive care (IC) therapy endpoints are traditionally defined as the IC mortality rate, hospital mortality rate, 30-day mortality rate combined with mechanical ventilation duration or the IC length of stay. Recently, the long-term survival rate and health-related quality of life (HRQL) outcomes have been identified as important alternative IC therapy endpoint markers [1]. Furthermore, post-traumatic stress disorder (PTSD), neuromuscular problems and activity impairment are considered long-term IC therapy complications [2, 3]. Previous studies regarding IC therapy survival have focused on total intensive care unit (ICU) patients, including post-operative patients with short ICU stays [4, 5], or have focused on specific populations, such as the elderly, cancer patients or mechanically ventilated patients [6–9]. Patient prognoses after long-term ICU stays (>72 h) are unknown. The main objective of this retrospective cohort study was to determine the long-term ICU-treated patient one-year mortality rate and to define its predictors. HRQL, place of living, healthcare use and possible long-term complications were assessed in the ICU survivors at 1, 2 and 5 years after ICU discharge.

## Methods

### Patients

This study was designed as a retrospective cohort study and was conducted at the 20-bed mixed closed-format ICU of the Onze Lieve Vrouwe Gasthuis in Amsterdam. Inclusion criteria was: an ICU stay of >72 h. Our study consisted of three cohorts, which were extracted from a dataset of all admitted ICU patients who were treated for more than 72 h between 1 January 2007 and 31 December 2007 (cohort 3), 1 June 2011 and 1 April 2012 (cohort 2) and 1 June 2012 and 1 October 2012 (cohort 1).

### Measurements

The death dates were obtained from the Dutch municipal records which registers each death and links it to a national database. Together with the hospital information system Xcare, the ICU survivors’ survival statuses were tracked. The ICU survivors that died within one year after ICU discharge, in all cohorts, were further analysed to identify any one-year mortality predictors. The ICU survivors were contacted and asked to participate in this study. A written informed consent was obtained from all participants. After permission was granted, they received two questionnaires: the RAND-36 item Health Survey (RAND-36) and a questionnaire that evaluated where the patients were residing and their healthcare use after hospital discharge. We also asked for written permission to retrieve information from their GPs regarding their long-term complications following IC therapy.

The RAND-36 is the Dutch language version of the SF-36, which is a validated and reliable generic HRQL measuring tool. This survey contains eight domains: physical functionality, limitations due physical health problems, bodily pain, general health perception, vitality, social functionality, limitations due to emotional health issues and general mental health. The bodily pain and general health perception domains are not comparable because of the differences between the RAND-36 and SF-36 [10]. For that reason, we did not use these domains in our analysis.

The questionnaire regarding home return and healthcare use after hospital discharge was developed by a team of experts composed of ICU nurses, intensivists and a clinical epidemiologist. The questionnaire consisted of five closed questions about where the patients were residing after hospital discharge and their healthcare use in the last 4 weeks.

The long-term complications questionnaire was developed following a literature research centred on long-term complications after IC therapy and a focus group meeting with GPs. It consisted of fifteen items and sub-questions regarding possible complications that can occur after IC therapy.

The following data were retrospectively collected for each patient from the Patient Data Management System (PDMS) (iMD-soft; Metavision, Tel aviv, Israël): gender, age at ICU admission, Body Mass Index, Acute Physiology and Chronic Health Evaluation score IV (APACHE), highest Sequential Organ Failure Assessment score (SOFA), length of stay (LOS) at ICU, LOS at hospital, mechanical ventilation duration (hours), type of admission (defined as elective surgery, emergency surgery and medical), presents of comorbidity, presence of comorbidity (defined as present/yes of not present/no), Continues Veno-Venous Hemofiltration (CVVH) during ICU treatment yes/no, sepsis in the first 24 h of the admission and readmission ICU after initial ICU treatment.

To assess the illness severity, we used the APACHE IV pm score [11]. To describe organ dysfunction/failure, we used the SOFA score [12].

This study was approved by the Onze Lieve Vrouwe Gasthuis Medical Ethics Committee in January 2013.

### Statistical analyses

Statistical analyses were performed using the SPSS Predictive Analytics Software 20.0 (SPSS Inc., Chicago, IL). All continuous and normally distributed data were reported as mean ± standard deviation. Ordinal and non normally distributed data were reported as median and interquartile ranges. Data were summarised by frequencies and percentages for the categorical variables. Predictors were a priori defined as age and APACHE IV pm score. One year survivors and non-survivors were compared by using chi-square test of Fisher’s exact test, Student’s *t* test or Mann–Whitney rank-sum test as appropriate.

We included Age and APACHE IV pm and all variables with *P* ≤ 0.20 in the univariable analysis in the multivariable Cox regression model, beginning the day of ICU discharge until death within one year. The final multivariable model was constructed by performing a backward stepwise manual selection method. The assumption of proportional hazards was graphically tested with a log minus log survival plot. The hazard ratio’s are reported with 95 % Confidence Interval (CI). A p value less than 0.05 was considered statistically significant.

The RAND-36 scale scores of the respondents were converted to a percentage ranging from 0 to 100, with higher values indicating higher levels of functioning and well-being. The mean RAND-36 scale scores were compared with those of an age-matched Amsterdam general population group with an independent *t*-test [13].

## Results

**Fig. 1:**
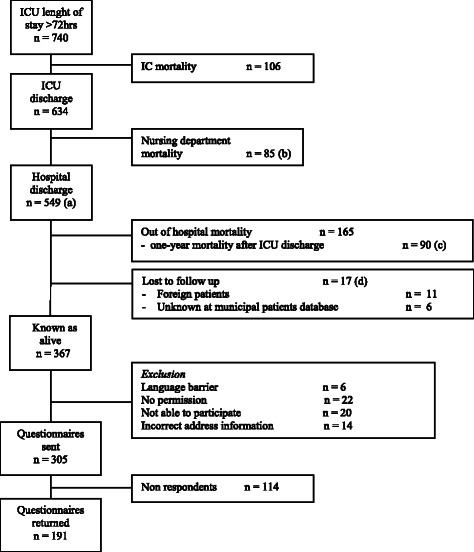
Flow diagram of the respondents. Survivors and non survivors one year after ICU discharge: survivors n (442) = a − c − d, non survivors n (175) = b + c

Seven hundred and forty patients were treated for more than 72 h in the ICU during the inclusion period. In total, 114 patients were included in cohort 1, 256 patients were included in cohort 2 and 370 patients were included in cohort 3. Of the 740 patients, 106 patients (14 %) died in the ICU and another 85 patients (12 %) died during their hospital stay after ICU discharge, resulting in a total of 191 in-hospital deaths (26 %) (Fig. 1).

### The one-year ICU survivor mortality rate

**Fig. 2:**
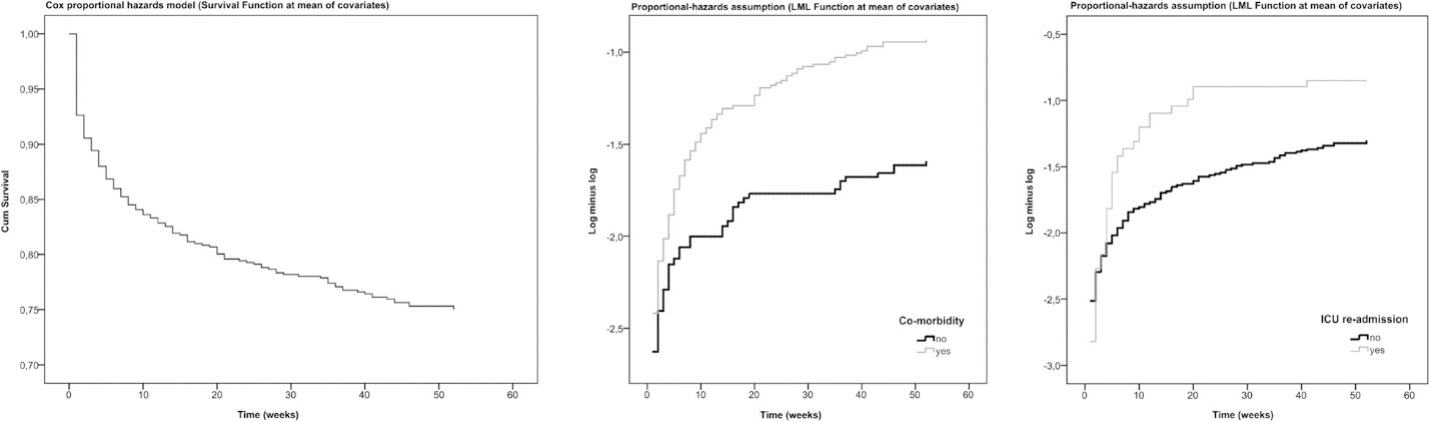
Cox regression analysis one-year mortality after ICU discharge

Of the 634 ICU survivors, 17 patients were lost to follow up, which left 617 patients for further analysis. Additionally, 175/617 patients (28 %) died within the first year after ICU discharge (Fig. 2). Of these 175 patients, 83 patients (47 %) died within the first week following ICU discharge. Moreover, 65/83 (78 %) died during their stay in our hospitals nursing ward. Additionally, 18/83 (22 %) died outside of our hospital; however, it is unknown whether they died at home, in another hospital or in a temporarily health facility. Of the group who died during the first week in the nursing ward, 7/65 (11 %) were discharged from the ICU with palliative care, 35/65 (54 %) were discharged with any treatment restrictions (from do-not-resuscitate orders to withholding of new intensive care therapy), 8/65 (12 %) died during ICU re-admission, and 12/65 (18 %) were discharged without an apparent course. The ICU survivor baseline characteristics are shown in Table 1. The age, APACHE IV pm, SOFA score, ICU length of stay, mechanical ventilation, admission type, comorbidity presence, CVVH and re-admission values significantly differed between the survivors and the non-survivors.

**Table 1:** Baseline characteristics ICU survivors

Characteristics	ICU survivors after 1 year *n* = 442	Non survivors after 1 year *n* = 175	*p*-value
Male/female^a^	273 (61,8)/169 (38,2)	112 (63,6)/64 (36,7)	.605^c^
Age (years)^b^	68 (58–75,3)	72 (63–77)	.002^d^
BMI at admission^b^	26,1 (23,4–29,8)	26,1 (23–30,4)	.967^d^
APACHE-IV PM score^b^	14,4 (4,1–38,3)	41,4 (21,9–71,9)	.0002^d^
Highest SOFA score^b^	8 (6–10)	9 (7–12)	.0002^d^
ICU Lenght of stay LOS (days)^b^	6 (5–10)	8 (5–11)	.011^d^
Hospital LOS (days)^b^	17 (11–31)	18 (11–33)	.620^d^
Mechanical Ventilation (hours)^b^	56 (16,8–115)	90 (38–181)	.000^d^
Admission type:^a^			.000^c^
Medical	182 (41,2)	112 (64)	
Elective Surgery	196 (44,3)	44 (25,7)	
Emergency Surgery	64 (14,5)	18 (10,3)	
Comorbidity^a^			.000^c^
yes	205 (46,4)	118 (67,4)	
no	237 (53,6)	57 (67,4)	
CVVH during ICU^a^	77 (17,4)	53 (30,3)	.001^c^
Sepsis at admission ICU^a^	66 (14,9)	25 (14,3)	.938^c^
Re-admission ICU after initial ICU- treatment^a^	47 (10,6)	32 (18,3)	.015^c^

*ICU* Int ensive Care Unit, *APACHE pm* Acute Physiology and Chronic Health Evaluation, predicted mortality, *SOFA* Sequential Organ Failure Assessment, *CVVH* Continuous Veno-Venous Hemofiltration

^a^Values are given as number and frequency (%)

^b^median (interquartile)

^c^Chi-Square

^d^M ann-Whitney

Multivariable Cox regression analysis identified four independent predicting factors of one-year mortality after ICU discharge, which included APACHE IV pm score (OR: 1.02; 95 % CI: 1.01–1.02), age at the time of ICU admission (OR: 1.02; 95 % CI: 1.00–1.03), re-admission after ICU discharge within the same hospital stay period (OR: 1.55; 95 % CI: 1.05–2.28), presence of comorbidities (OR: 1.86; 95 % CI: 1.35–2.56), (Table 2). The survival curve at mean of the covariates for time to one-year mortality is shown in Fig. 2.

**Table 2:** Cox regression analysis: factors independently associated with 1-year mortality after ICU discharge

	95 % CI			
Variables	HR	Lower	Higher	Significance (*p*-value)
AGE	1,017	1,004	1,031	0,010
APACHE IV pm	1,018	1,013	1,023	0,000
Co-morbidity	1,857	1,348	2,559	0,000
ICU re-admission	1,555	1,055	2,285	0,026

*HR* Hazard Ratio, *CI* Confidence Interval ICU, Intensive Care U nit, *APACHE IV pm* Acute Physiology and Chronic Health Evaluation, IV predicted mortality

### Health related quality of life

The HRQL questionnaire was sent to 305 survivors of the three cohorts (1, 2 and 5 years after ICU discharge), and 191 of these patients (63 %) completed the questionnaire (Fig. 1). The RAND-36 scores are summarised in Table 3. Of all the cohorts, the mean values of the six analysed domains (physical functionality, physical role, social functionality, mental health, emotional well-being and vitality) were significantly lower than in the Amsterdam control group.

**Table 3:** Scores on the RAND-36

	Cohort 1			Cohort 2			Cohort 3			Control Group Amsterdam	
		T score^b^	T score^b^		T score^b^	T score^b^		T score^b^	T score^b^	Overall	Age group (60–69)
Domains	*n* = 40^a^	$\overline{\mathrm{x}}$	a	*n* = 69^a^	$\overline{\mathrm{x}}$	a	*n* = 82^a^	$\overline{\mathrm{x}}$	a	$\overline{\mathrm{x}}$	a
										*n* = 4172^a^	*n* = ±354^a^
Physical functioning	52.5 ± 30.4	−6.80^c^	−4.64^c^	47.8 ± 29.9	−10.3^c^	−7.40^c^	62.6 ± 27.8	−7.38^c^	−4.00^c^	85.2 ± 23.1	74.8 ± 27.1
Role physical	44.7 ± 42.8	−5.01^c^	−4.07^c^	45.9 ± 41.0	−6.72^c^	−5.42^c^	54.5 ± 43.9	−5.03^c^	−3.72^c^	79.5 ± 35.4	73.0 ± 39.7
Social functioning	65.9 ± 29.0	−4.18^c^	−4.14^c^	67.8 ± 25.8	−5.53^c^	−5.46^c^	74.1 ± 26.2	−3.79^c^	−3.72^c^	85.1 ± 21.5	84.9 ± 22.9
Mental health	70.3 ± 20.5	−1.73	−2.62^c^	70.5 ± 20.7	−2.16^c^	−3.32^c^	73.5 ± 17.6	−1.25	−2.73^c^	75.9 ± 17.0	78.8 ± 17.4
Role emotional	58.3 ± 44.6	−3.33^c^	−3.64^c^	63.1 ± 41.8	−3.88^c^	−4.30^c^	73.9 ± 40.4	−2.00^c^	−2.48^c^	83.1 ± 32.7	85.3 ± 31.1
Vitality	54.5 ± 21.2	−4.20^c^	−4.68^c^	54.1 ± 20.0	−6.00^c^	−6.64^c^	57.8 ± 21.3	−4.52^c^	−5.19^c^	68.6 ± 19.2	70.2 ± 20.4

*Cohort 1*1 year after ICU discharge, *Cohort 2* 2 year after ICU discharge, *Cohort 3* 5 year after ICU discharge

^a^The scores, which have a possible range of 0 to 100, are given as the mean and the standard deviation

^b^The T score represents the comparison of the scores of a cohort and those of Amsterdam study: mean age ($\overline{\mathrm{x}}$) and Amsterdam study: age group 60–69 year (a)

^c^Significance

### The place of residence and healthcare use after ICU discharge

The questionnaire regarding home return and healthcare use after hospital discharge was sent to the same 3 cohorts of 305 ICU survivors. A total of 190 (62 %) of the survivors completed the questionnaire. 85/190 respondents (45 %) did not return to their home situation after ICU discharge, 47/85 (55 %) of these respondents were transferred to a rehabilitation facility. Additionally, 68/105 (65 %) of the respondents who did return to their home situation were living independently. At the time of this research, 180 (95 %) of the respondent ICU survivors had a self-sufficient current living situation, which included self-sufficiency with home- and/or informal care. Additionally, 78 respondents (41 %) had an appointment with their GP, 82 (43 %) met with a medical specialist and 28 (15 %) met with a physiotherapist within the last 4 weeks.

### Long-term complications after an ICU stay

A total of 162/191 (85 %) respondents gave permission to retrieve additional information from their GPs, and 95 GPs (59 %) responded to our questionnaire. The possible long-term IC therapy complications that were most frequently reported included general health complaints about exercise tolerance (*n* = 42, 44 %), general health complaints about chronic fatigue (*n* = 35, 37 %), chronic renal insufficiency (*n* = 19, 20 %), an inability to return to pre-ICU admission daily activities (*n* = 17, 18 %), processing problems of relatives (*n* = 14, 15 %), anaemia (*n* = 14, 15 %) and congestive heart failure (*n* = 13, 14 %).

## Discussion

The one-year post-ICU discharge mortality rate of the patients who were treated for more than 72 h in the ICU was 28 %, of which a large proportion died within the first week after ICU discharge. The one-year mortality rates were related to increased age at the time of ICU admission, high APACHE IV-predicted mortality scores, presence of comorbidities and ICU re-admission. Additionally, the ICU survivors had a significantly lower HRQL compared to a control group and a large number of these patients (45 %) did not return directly to their home situations after hospital discharge. Further, the most often reported long-term ICU complications were decreased exercise tolerance, chronic fatigue, processing family-related issues, an inability to return to pre-ICU admission daily activities, anaemia, renal insufficiency and congestive heart failure.

This research article is the first report of one-year post-ICU discharge mortality rates in long-term treated ICU patients. Most of the previous reports included the one-year mortality rate of the total ICU patient population. We choose to observe the group of patients with a longer ICU stay, as they most likely suffered from a higher illness severity level, making them at risk for long-term complications. The mortality rates were, as expected, lower (5–13 %) than in the previous studies that included all IC patients, due to the presence of a large cohort of post-surgical patients with low APACHE pm scores [14–16]. In addition to the differences in the patient populations (total versus long-term stay ICU patients), the above-mentioned studies also differed in design. Our study reports the one-year post-ICU discharge mortality rate, whereas the others used the one-year post-hospital discharge mortality rates. The period after an ICU discharge, in particular, gives the complete picture of the situation after ICU therapy, which was of specific interest in our study, as explained in the next paragraph.

A large proportion of the patients (47 %) died within the first week of ICU discharge, which is comparable to the Araújo et al. study [17]. A possible explanation for this finding could be the decision to withhold or withdraw life-sustaining treatments during a patient’s ICU stay as the most powerful predictor of post-IC mortality, which was reported previously [18]. Though the majority of patients who died within the first week following ICU discharge were discharged with any restriction for further medical therapy, the number of patients discharged with palliative care was limited. The patients with medical therapy restrictions are most likely at higher risk for in-hospital death due to their underlying diseases. Nevertheless, other possible explanations include inappropriate triage before ICU admission, ICU case-mix (e.g., increased age, several comorbidities and illness severity), inappropriate post-ICU care and lack of ICU capacity [19].

The one-year mortality predictors included increased age at the time of ICU admission, a high APACHE IV pm score, presence of comorbidities and ICU re-admission. Most of these predictors have been previously reported, including age [5, 14, 16, 20, 21], comorbidity [14, 16, 21] and illness severity [5, 6, 16, 18]. Additionally, a ‘sepsis’ diagnosis was found in previously studies to be associated with an increased mortality rate in the first year after an ICU stay [22]. This association was not observed in our study, most likely because of the small population size and the definition of sepsis. We only had valid data of the incidence of sepsis in the first 24 h of the ICU admission. Notably, re-admission was not previously reported.

The HRQL in the ICU survivors who were treated for more than 72 h was evaluated using the RAND-36. This study suggests that at three different cohorts after ICU discharge, the ICU survivors had significantly lower scores in six HRQL domains compared with the HRQL of the age-matched Amsterdam study population group. No studies were found that used the same follow-up period and inclusion/exclusion criteria that we used. Two systematic reviews used many studies that differ in designs, patient populations, HRQL instruments, follow-up times and response rates, although their overall findings are consistent with our findings [23, 24]. Additionally, a higher illness severity and a higher chronic health burden may lead to a diminished HRQL [25, 26].

Many patients needed additional institutional and home care after long-term IC therapy hospital discharge, and a minority of the patients directly returned home. This differs from a previous Australian study in which a majority of the patients (82 %) returned to their home after hospital discharge [27]. Both studies are difficult to compare because of the differences in the organisation of the healthcare systems between our countries. Moreover, there are important post-hospital healthcare organisation differences for the patients who require long-term care between the Netherlands and Australia [28]. The findings in our study could be related to the wide gap between hospital and home care after IC therapy, the ICU case-mix, and especially, the lack of informal care culture in the Netherlands. Finally, 97 % of the patients returned to their home again, with or without home and informal care.

In the extensive long-term ICU complication survey, which were reported by the GPs of the long-term ICU-treated survivors, the following items were reported: decreased exercise tolerance, chronic fatigue, processing problems of relatives, an inability to return to pre-ICU daily activities, anaemia, renal insufficiency and congestive heart failure. Surprisingly, PTSD, the most well-known long-term ICU stay complication, was only reported in a minority of the patients. This was most likely due to a difference in the PTSD diagnoses between the GPs and the studies that reported PTSD as common post-ICU complication [2]. Additionally, it is possible that the ICU survivors that would be diagnosed with PTSD using a validated PTSD questionnaire, but the patient may not visit the GPs with these problems because of fear, shame or did not consider it a problem. More research regarding long-term complications among ICU survivors is needed to anticipate which interventions concern post-ICU care.

### Limitations

We acknowledge certain potential limitations in our study. First, as a result of the retrospective observational cohort design, we could not compare the RAND-36 results of our three cohorts to discern associations regarding the HRQL over time. In a prospective cohort study, we would expect an HRQL improvement over time because when a patient gets older or acquires a chronic disease, he changes his internal standards and values and conceptualisation of HRQL [29]. This could be either because the patients become accustomed to their illness or chronic disease or because their expectations about their HRQL have changed [30]. Furthermore, regarding the high first week post-ICU discharge mortality rate analysis, we only had information for the patients who stayed in our hospital. We had no access to the data of the patients who were transferred to other hospitals. Third, to understand an ICU patient HRQL, it is important to know the patient’s baseline HRQL before hospitalisation. Three methods are considered in estimating the baseline HRQL status: 1) the age- and sex-matched population normal values; 2) the patient/proxy reports; and 3) the patient’s retrospective recall [31]. Because of the retrospective design of this study, we chose to use an age-matched population normal value. Additionally, we realised that this method and the other abovementioned methods do not provide the exact baseline HRQL of the ICU survivors. First, previous studies showed that ICU patients have lower baseline HRQL levels than the normal population [26, 30]. Second, a patient’s retrospective baseline HRQL assessment may suffer from recall bias [32]. Lastly, some studies showed a poor to fair agreement of proxy versus the patient HRQL assessments [26, 33]. Nonetheless, we had to use one of these methods in the absence of a better alternative, but we noticed that there was no recent RAND-36 score with which to compare our results. Third, the long-term complication questionnaire was designed as a screening instrument to identify long-term complications for further research and, therefore, might be biased by the way the GPs measured post-ICU problems. Additionally, not all ICU survivors may have visited their GPs with their post-IC therapy complaints. Finally, the assumption of proportional hazards may be violated because the curves for re-admission crossed at the beginning of the graph (Fig. 2). This may be due to the relatively high mortality in the first week after ICU discharge. Future research with a larger sample size should take this into account.

## Conclusions

Of the long-term (>72 h) treated ICU patients, 28 % died within one year after ICU discharge. Furthermore, half of these patients died in the first week following ICU discharge. Increased age at the time of ICU admission, a high APACHE IV-predicted mortality score, presence of co-morbidities and re-admission were associated with an increased one-year mortality rate. Additionally, the ICU survivors reported a lower HRQL, of which a minority directly returned home after hospital discharge, and the most commonly scored complications were decreased exercise tolerance, chronic fatigue and processing problems of relatives.
